# Supplementary material for: ACS-20/FATP4 mediates the anti-ageing effect of dietary restriction in C. elegans
Source: Nat Commun. 2023 Nov 24;14:7683. doi: 10.1038/s41467-023-43613-4 (PMC10673863; doi:10.1038/s41467-023-43613-4)
Supplement: Supplementary file 1 — Supplementary Information [file 41467_2023_43613_MOESM1_ESM.pdf]

Supplemental materials for

ACS-20/FATP4 mediates the anti-ageing effect of dietary restriction in *C. elegans*

Zi Wang[1], Lina Zou[1], Yiyan Zhang[1,2], Mengnan Zhu[3], Shuxian Zhang[3], Di Wu[4], Jianfeng Lan[5], Xiao Zang[1,2], Qi Wang[1,2], Hanxin Zhang[1], Zixing Wu[1], Huanhu Zhu[3], Di Chen[1,2,6] ✉

[1] Model Animal Research Center of Medical School, MOE Key Laboratory of Model Animals for Disease Study, Nanjing University, Nanjing, Jiangsu 210061, China

[2] Zhejiang University-University of Edinburgh Institute, Zhejiang University School of Medicine, Haining, 314400, China

[3] School of Life Science and Technology, ShanghaiTech University, Shanghai, 201210, China

[4] Institute of Drug Discovery and Development, Center for Drug Safety Evaluation and Research, Zhengzhou University, Zhengzhou, Henan 450001, China

[5] Affiliated Hospital of Guilin Medical University, Guilin, Guangxi 541001, China

[6] Department of Colorectal Surgery, The Second Affiliated Hospital, Zhejiang University School of Medicine, Hangzhou, Zhejiang 310058, China

✉ email: dic@intl.zju.edu.cn, chendi@nju.edu.cn

Supplementary Fig. 1 (related to Fig. 1)

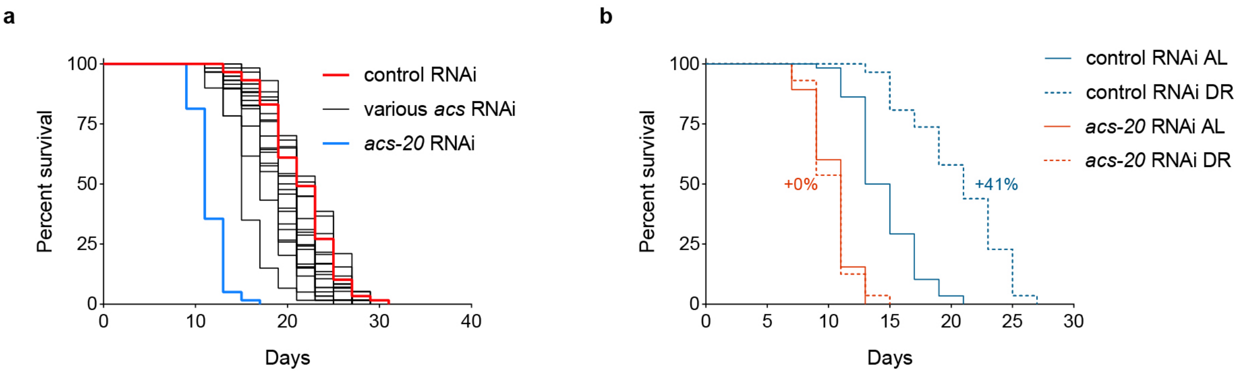

Supplementary Fig. 1 (related to Fig. 1) *acs-20* RNAi knockdown blocks the bacterial food dilution-induced lifespan extension. a Survival curves of N2 animals treated with the control or various *acs* genes RNAi during development and then subjected to diluted bacterial food (1.0 x 10^9 cfu / ml) during adulthood. control vs. *acs-20* RNAi, $p < 0.0001$ (log-rank test). **b** Survival curves of N2 animals treated with the control or *acs-20* RNAi during development and then subjected to AL (1.0 x 10^{11} cfu / ml) or DR (1.0 x 10^9 cfu / ml) feeding during adulthood. control RNAi AL vs. DR, $p <$ 0.0001; *acs-20* RNAi AL vs. DR, $p = 0.8615$ (log-rank tests). Percentages indicate changes in mean lifespan induced by DR. Source data are provided as a Source Data file.

Supplementary Fig. 2 (related to Fig. 2)

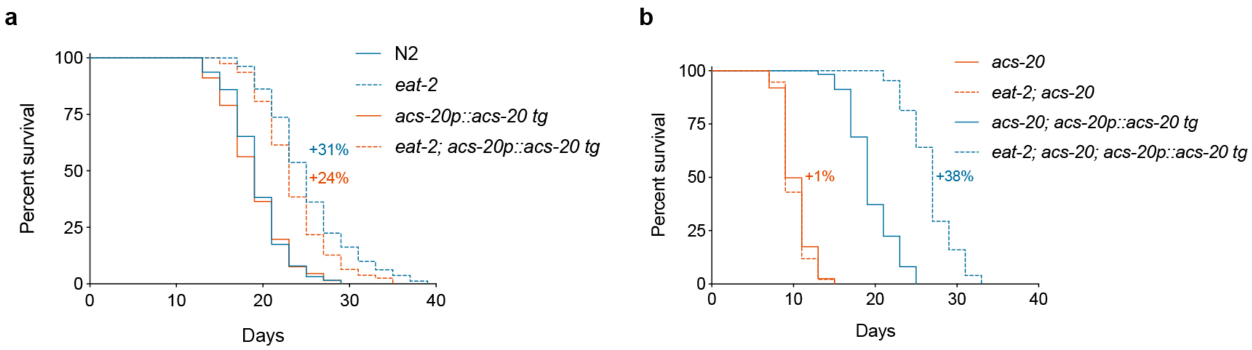

Supplementary Fig. 2 (related to Fig. 2) Transgenic rescue of the *acs-20* mutant for its defect in DR-induced lifespan extension. a Survival curves of N2 and *eat-2* mutants with or without the *acs-20* transgene driven by the native *acs-20* promoter. N2 vs. *eat-2*, $p < 0.0001$; *acs-20p::acs-20 tg* vs. *eat-2; acs-20p::acs-20 tg*, $p < 0.0001$ (log-rank tests). **b** Survival curves of *acs-20* and *eat-2; acs-20* mutants with or without the *acs-20* transgene driven by the native *acs-20* promoter. *acs-20* vs. *eat-2; acs-20*, $p = 0.4758$; *acs-20*; *acs-20p::acs-20 tg* vs. *eat-2; acs-20; acs-20p::acs-20 tg*, $p < 0.0001$ (log-rank tests). Percentages indicate changes in mean lifespan induced by the *eat-2* mutation. Source data are provided as a Source Data file.

Supplementary Fig. 3 (related to Fig. 3)

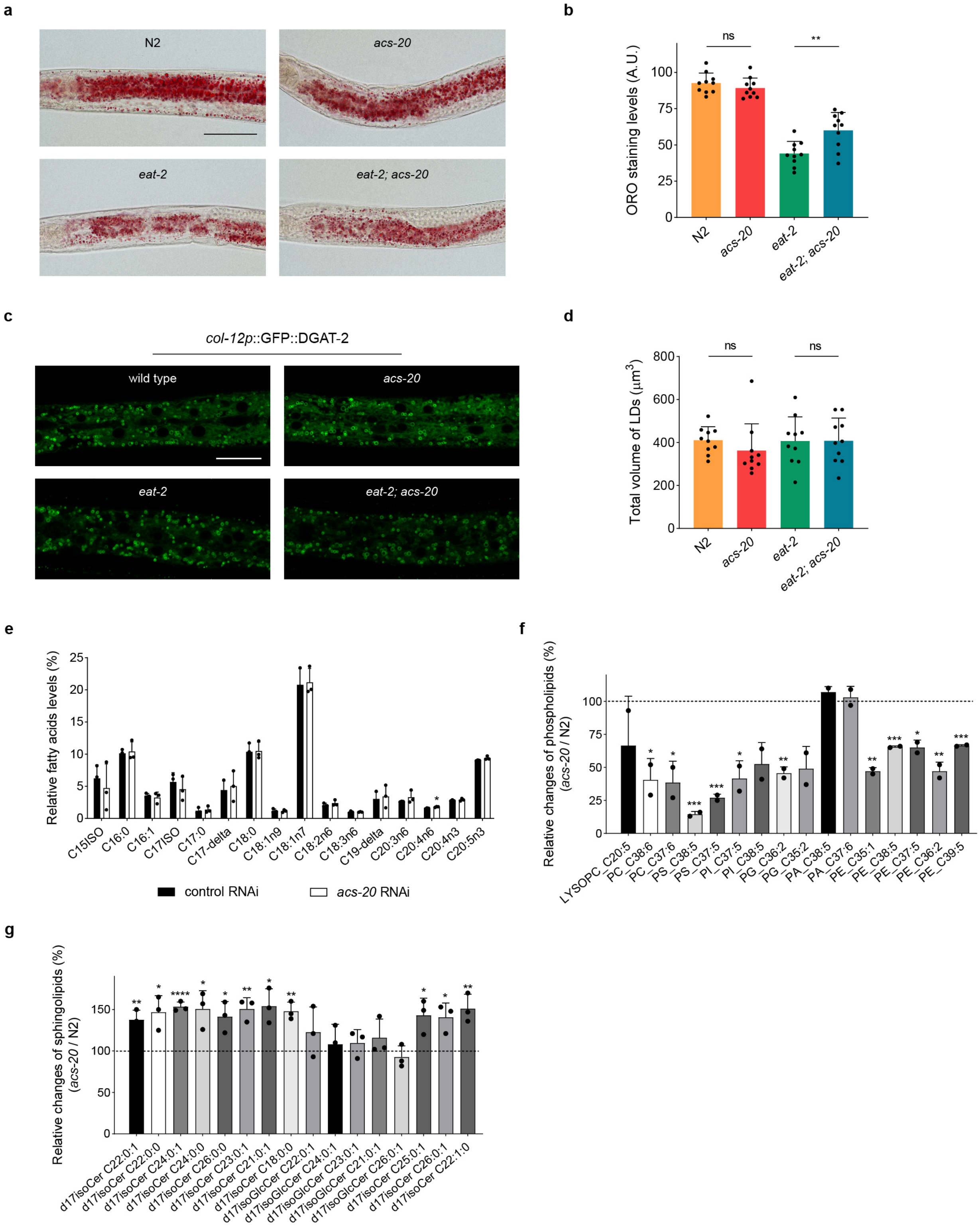

Supplementary Fig. 3 (related to Fig. 3) Effects of the *acs-20* mutation on lipid metabolism. a-b Representative Oil Red O staining images (**a**) and quantification of the

staining signal (**b**) in N2, *acs-20*, *eat-2*, and *eat-2; acs-20* backgrounds. Data are represented as mean ± SD from 10 animals. ns, $p = 0.8414$; **, $p < 0.01$ (One-way ANOVA with Tukey's multiple comparison test). Scale bar, 50 μm. **c-d** Representative photographs of the *col-12p*::GFP::DGAT-2 expression in the epidermis (**c**) and quantification of epidermal lipid droplets (**d**) in wild-type, *acs-20*, *eat-2*, and *eat-2; acs-20* backgrounds. Data are represented as mean ± SD from 10 animals. ns, $p = 0.7308$ (N2 vs. *acs-20*); $p > 0.9999$ (*eat-2* vs. *eat-2; acs-20*) (One-way ANOVA with Tukey's multiple comparison test). Scale bar, 20 μm. **e** Effects of the *acs-20* RNAi treatment on fatty acids in wild-type animals. *, $p < 0.05$ (two-tailed t-test). **f** Effects of the *acs-20* mutation on phospholipids. *, $p < 0.05$; **, $p < 0.01$; ***, $p < 0.001$ (two-tailed t-tests). **g** Effects of the *acs-20* mutation on sphingolipids. *, $p < 0.05$; **, $p < 0.01$; ****, $p < 0.0001$ (two-tailed t-tests). Samples without asterisks on top of the bars are not significantly different from the control ($p > 0.05$, two-tailed t-tests). Source data are provided as a Source Data file.

Supplementary Fig. 4 (related to Fig. 3 and Fig. 4)

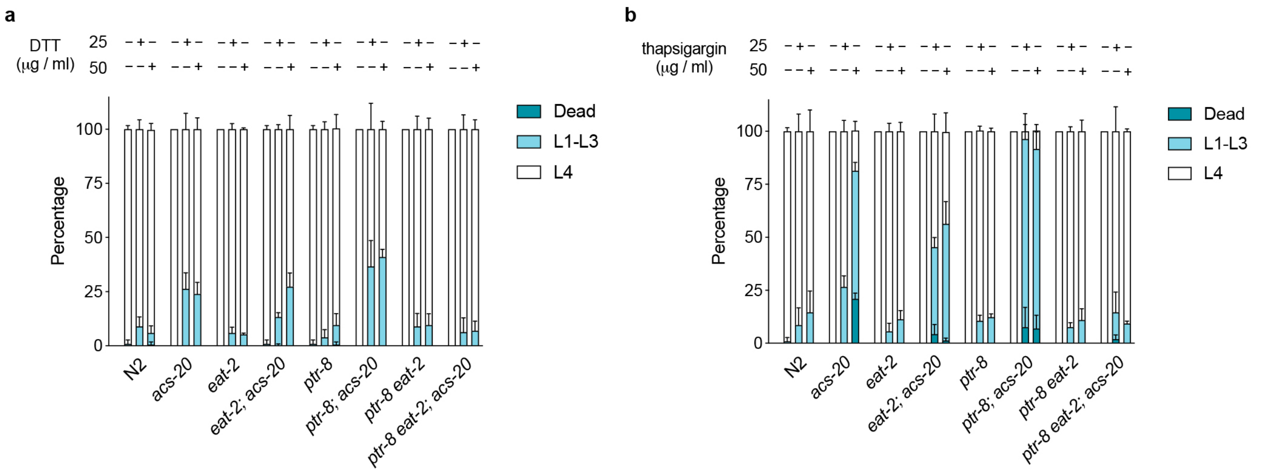

Supplementary Fig. 4 (related to Fig. 3 and Fig. 4) The *acs-20* mutation causes PTR-dependent ER stress sensitivity. a-b Percentages of N2, *acs-20*, *eat-2*, *eat-2; acs-20*, *ptr-8*, *ptr-8; acs-20*, *ptr-8 eat-2*, and *ptr-8 eat-2; acs-20* mutant animals that died (dead), showed developmental arrest (L1-L3) or completed development (L4) upon treatment with 0, 25 or 50 μg / ml of DTT (**a**) or thapsigargin (**b**). Data are represented as mean ± SD based on three biological replicates. For animals that completed development upon DTT or thapsigargin treatments, *eat-2* vs. *eat-2; acs-20*, $p < 0.0001$; *eat-2; acs-20* vs. *ptr-8 eat-2; acs-20*, $p < 0.0001$ (Two-way ANOVA with Tukey's multiple comparison tests). Source data are provided as a Source Data file.

Supplementary Fig. 5 (related to Fig. 5)

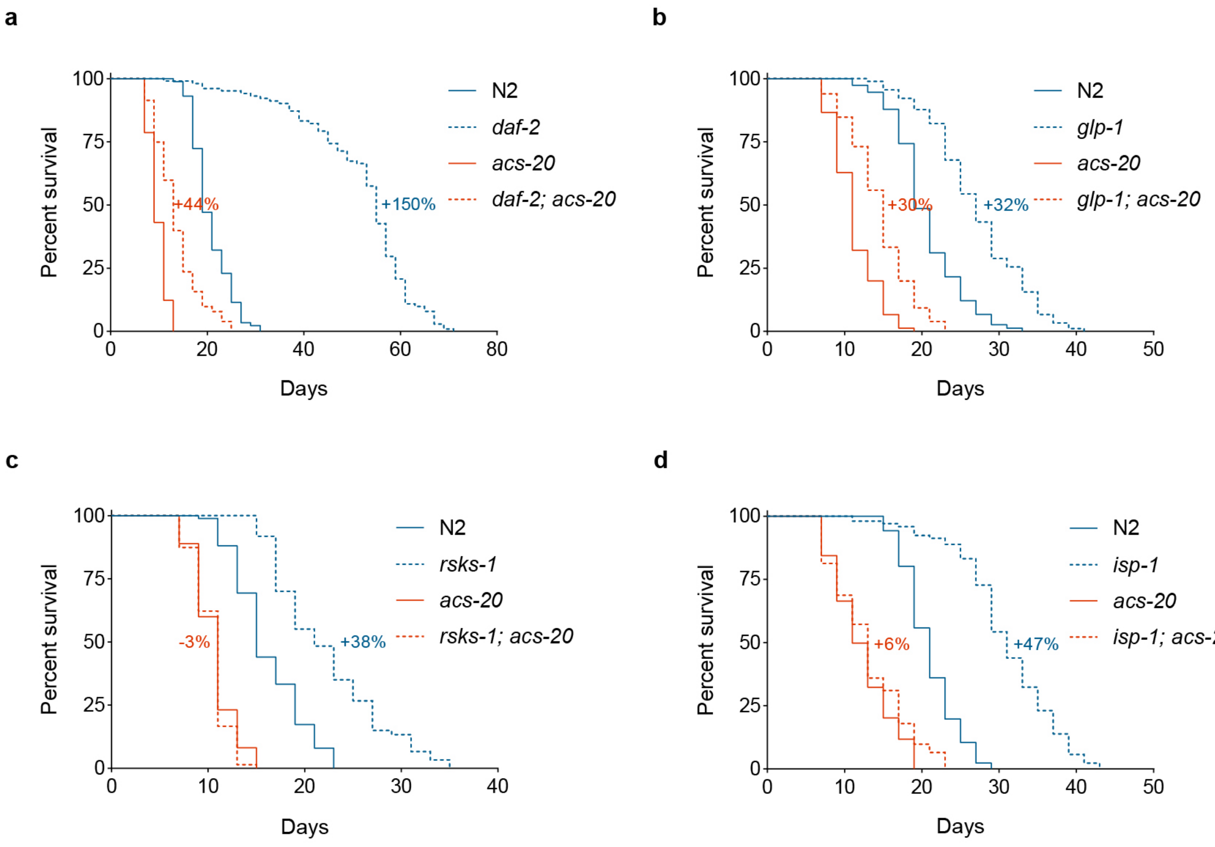

Supplementary Fig. 5 (related to Fig. 5) Effects of the *acs-20* mutation on various long-lived mutants. a Survival curves of N2, *daf-2*, *acs-20*, and *daf-2; acs-20* animals. N2 vs. *daf-2*, $p < 0.0001$; *acs-20* vs. *daf-2; acs-20*, $p < 0.0001$ (log-rank tests). **b** Survival curves of N2, *glp-1*, *acs-20*, and *glp-1; acs-20* animals. N2 vs. *glp-1*, $p < 0.0001$; *acs-20* vs. *glp-1; acs-20*, $p < 0.0001$ (log-rank tests). **c** Survival curves of N2, *rsks-1*, *acs-20*, and *rsks-1; acs-20* animals. N2 vs. *rsks-1*, $p < 0.0001$; *acs-20* vs. *rsks-1; acs-20*, $p = 0.4374$ (log-rank tests). **d** Survival curves of N2, *isp-1*, *acs-20*, and *isp-1; acs-20* animals. N2 vs. *isp-1*, $p < 0.0001$; *acs-20* vs. *isp-1; acs-20*, $p = 0.1793$ (log-rank tests). Percentages indicate changes in the mean lifespan induced by various long-lived mutants with or without ACS-20. Source data are provided as a Source Data file.

Supplementary Methods (related to Supplementary Fig. 3)

Lipid staining by Oil Red O

L4 animals were collected and fixed in 1% formaldehyde and frozen at -80°C. The samples were subject to three cycles of freezing and thawing with dry ice / ethanol bath and a stream of warm water, respectively. After washed twice with the S buffer, animals were incubated in the Oil red O (3 mg / ml) solution for 30 minutes at the room temperature. Animals were then washed with the S buffer and incubated on ice for 15 min. Images were taken using a Nikon Eclipse Ni-U microscope equipped with a DS-Fi2 color CCD. Mean intensity of Oil Red O signal in the second pair of intestinal cells was quantified using the ImageJ software.

Fluorescence imaging and quantification of epidermal lipid droplets

The *col-12* promoter driving GFP::DGAT-2 in L4 animals were imaged with a Zeiss LSM880 confocal microscope equipped with an Airyscan module. Animals were imaged by a 40X/1.3W objective with a zoom value equal to 2, and GFP signals were excited by a 488 nm laser and collected with a BP 495-550 + LP 570 emission filter. Optical sections were taken at 0.45 µm intervals and ten focal planes in total (4.05 µm in z axis) were used for all projected fluorescence images, which were then exported to Imaris 9 (Bitplane) for processing and 3D reconstruction. The diameter of each LD present in the second intestinal segment was fitted using the spot function in Imaris.

Lipid analysis by gas chromatography and mass spectrometry.

GC-MS samples were prepared as previously described[1]. Lipid extracts were dissolved in 1 ml of methanol with 1 mM formic acid and subjected to quantitative lipid analysis using a 4000 Q-Trap mass spectrometer (AB Sciex). Samples were infused at a flow rate of 8 µl/min using a Harvard Apparatus syringe pump (Harvard Apparatus).

Quantification of lipid component by UPLC/QTRAP-MS.

Around 5-30 mg of L4 larvae were collected for total lipid extractions as previously described[1, 2, 3]. The lipid analysis was performed using the SHIMADZU LC30A UHPLC

system equipped with the Luna®3μm silica column (100Å LC column 150 X 2mm) and the SCIEX Qtrap6500. The total lipid extractions were separated by the binary mobile phase, with a gradient from phase A (isopropanol/hexane/100 mM ammonium acetate 58/40/2) to phase B (isopropanol/hexane/100 mM ammonium acetate 50/40/10) being used. The contents of indicated phospholipids, sphingolipids, and sphingomyelin were quantified by multiple reaction monitoring (MRM) and normalized with externally supplied d18:1/C4-Ceramide or endogenous 15-methylhexadecanoic acid (C17ISO). Data acquisition and processing were performed by the Analyst and Peakview (Sciex).

Supplementary References

1. Zhu H, Shen H, Sewell AK, Kniazeva M, Han M. A novel sphingolipid-TORC1 pathway critically promotes postembryonic development in Caenorhabditis elegans. *Elife* **2**, e00429 (2013).

2. Bligh EG, Dyer WJ. A rapid method of total lipid extraction and purification. *Can J Biochem Physiol* **37**, 911-917 (1959).

3. Lin L*, et al.* Functional lipidomics: Palmitic acid impairs hepatocellular carcinoma development by modulating membrane fluidity and glucose metabolism. *Hepatology* **66**, 432-448 (2017).
