# Supplementary material for: ACS-20/FATP4 mediates the anti-ageing effect of dietary restriction in C. elegans
Source: Nat Commun. 2023 Nov 24;14:7683. doi: 10.1038/s41467-023-43613-4 (PMC10673863; doi:10.1038/s41467-023-43613-4)
Supplement: Supplementary file 3 — Description of Additional Supplementary Information [file 41467_2023_43613_MOESM3_ESM.pdf]

Description of Additional Supplementary Files

Supplementary data legends

Supplementary Data 1. Lists of the differentially expressed genes caused by the *acs-20* mutation. The data were calculated from three biological repeats of the wild-type N2, *acs-20*, *eat-2*, and *eat-2; acs-20* mutant animals via DESeq2. The adjusted *p* values were calculated using Wald tests with Bonferroni corrections.

Supplementary Data 2. List of *C. elegans* strains used.

Supplementary Data 3. List of oligos used.
