# Supplementary material for: ACS-20/FATP4 mediates the anti-ageing effect of dietary restriction in C. elegans
Source: Nat Commun. 2023 Nov 24;14:7683. doi: 10.1038/s41467-023-43613-4 (PMC10673863; doi:10.1038/s41467-023-43613-4)
Supplement: Supplementary file 7 — Reporting Summary [file 41467_2023_43613_MOESM7_ESM.pdf]

nature portfolio

Corresponding author(s): Di Chen

Last updated by author(s): Nov 5, 2023

Reporting Summary

Nature Portfolio wishes to improve the reproducibility of the work that we publish. This form provides structure for consistency and transparency in reporting. For further information on Nature Portfolio policies, see our Editorial Policies and the Editorial Policy Checklist.

Statistics

For all statistical analyses, confirm that the following items are present in the figure legend, table legend, main text, or Methods section.

n/a	Confirmed	
☐	☒	The exact sample size (*n*) for each experimental group/condition, given as a discrete number and unit of measurement
☐	☒	A statement on whether measurements were taken from distinct samples or whether the same sample was measured repeatedly
☐	☒	The statistical test(s) used AND whether they are one- or two-sided<br>*Only common tests should be described solely by name; describe more complex techniques in the Methods section.*
☒	☐	A description of all covariates tested
☒	☐	A description of any assumptions or corrections, such as tests of normality and adjustment for multiple comparisons
☐	☒	A full description of the statistical parameters including central tendency (e.g. means) or other basic estimates (e.g. regression coefficient) AND variation (e.g. standard deviation) or associated estimates of uncertainty (e.g. confidence intervals)
☐	☒	For null hypothesis testing, the test statistic (e.g. *F*, *t*, *r*) with confidence intervals, effect sizes, degrees of freedom and *P* value noted<br>*Give P values as exact values whenever suitable.*
☒	☐	For Bayesian analysis, information on the choice of priors and Markov chain Monte Carlo settings
☒	☐	For hierarchical and complex designs, identification of the appropriate level for tests and full reporting of outcomes
☒	☐	Estimates of effect sizes (e.g. Cohen's *d*, Pearson's *r*), indicating how they were calculated

Our web collection on statistics for biologists contains articles on many of the points above.

Software and code

Policy information about availability of computer code

Data collection	1. Images in Fig. 1d, Fig. 2a, 2b, Fig. 4e, and Supplementary Fig. 3c were taken using the ZEN 3.6 software (Carl Zeiss). Images in Fig. 3e and Supplementary Fig. 3a were taken using the NIS-Elements Software (Nikon). Images in Fig. 5a, b, e were taken using the LAS Life Science Microscope Software (Leica).<br>2. Western blot images were scanned with the Tanon-5200 Chemiluminescent Imaging System.<br>3. Mass spectrum data were collected using the Analyst and Peakview (AB SCIEX).<br>4. RT-qPCR data were collected using the Lightcycler 96 (Roche).
Data analysis	1. Results of lipid stained by Oil Red O (Supplementary S3a), fluorescence intensities (Fig. 3e), Western blots (Fig. 1h), and filter trap data (Fig. 4h) were quantified with ImageJ.<br>2. RNA-seq data were aligned to the C. elegans genome (WS220) using the spliced-junction mapper TopHat249. Aligned reads were counted per gene using the python script HTseq50. Differentially expressed genes were determined via DESeq2.<br>3. GO analyses was performed using Metascape (https://metascape.org/).<br>4. Mass spectrum data acquisition and processing were carried out via the Analyst and Peakview (Sciex)<br>5. DGAT-2::GFP fluorescence images (Supplementary S3c) were exported to Imaris 9 (Bitplane) for processing and 3D reconstruction. The diameter of each LD present in the second intestinal segment was fitted using the spot function in Imaris.<br>6. Statistical analyses were performed using the Graphpad Prism.

For manuscripts utilizing custom algorithms or software that are central to the research but not yet described in published literature, software must be made available to editors and reviewers. We strongly encourage code deposition in a community repository (e.g. GitHub). See the Nature Portfolio guidelines for submitting code & software for further information.

Data

Policy information about availability of data

All manuscripts must include a data availability statement. This statement should provide the following information, where applicable:

- Accession codes, unique identifiers, or web links for publicly available datasets
- A description of any restrictions on data availability
- For clinical datasets or third party data, please ensure that the statement adheres to our policy

The mRNA-Seq data have been deposited at the NCBI under the accession number GSE125718 (https://www.ncbi.nlm.nih.gov/geo/query/acc.cgi?acc=GSE125718). The source data have been provided in the Source Data file.

Research involving human participants, their data, or biological material

Policy information about studies with human participants or human data. See also policy information about sex, gender (identity/presentation), and sexual orientation and race, ethnicity and racism.

Reporting on sex and gender	NA
Reporting on race, ethnicity, or other socially relevant groupings	NA
Population characteristics	NA
Recruitment	NA
Ethics oversight	NA

Note that full information on the approval of the study protocol must also be provided in the manuscript.

Field-specific reporting

Please select the one below that is the best fit for your research. If you are not sure, read the appropriate sections before making your selection.

☒ Life sciences ☐ Behavioural & social sciences ☐ Ecological, evolutionary & environmental sciences

For a reference copy of the document with all sections, see nature.com/documents/nr-reporting-summary-flat.pdf

Life sciences study design

All studies must disclose on these points even when the disclosure is negative.

Sample size	Samples sizes were determined by standard protocols and procedures in the filed and they were shown in figure legends and source data. The sample sizes that we used are equal or above the average of the acceptable standard.
Data exclusions	No data were excluded.
Replication	All experiments in this study were independently repeated at least twice, in most cases more than three times, with consistent results.
Randomization	In each experiment, worms were randomly selected for analysis from a large population of animals.
Blinding	The investigators were not blinded during data collection because large numbers of strains under different conditions such as RNAi treatments, food, and temperature, were used in the experiments. Most experiments have been independently carried out by multiple authors.

Reporting for specific materials, systems and methods

We require information from authors about some types of materials, experimental systems and methods used in many studies. Here, indicate whether each material, system or method listed is relevant to your study. If you are not sure if a list item applies to your research, read the appropriate section before selecting a response.

Materials & experimental systems

n/a	Involved in the study
☐	☒ Antibodies
☒	☐ Eukaryotic cell lines
☒	☐ Palaeontology and archaeology
☐	☒ Animals and other organisms
☒	☐ Clinical data
☒	☐ Dual use research of concern
☒	☐ Plants

Methods

n/a	Involved in the study
☒	☐ ChIP-seq
☒	☐ Flow cytometry
☒	☐ MRI-based neuroimaging

Antibodies

Antibodies used	monoclonal anti-FLAG (Sigma 1804, dilution 1:10,000)<br>monoclonal anti-Tubulin Alpha (Sigma T6074, dilution 1:10,000)<br>anti-GFP (AMSBIO TP401, dilution 1:500)<br>goat anti-mouse IgG (H+L) HRP (Bioworld BS12478, dilution 1:10,000)
Validation	The anti-FLAG antibody has been validated for immunoblotting in C. elegans (PMID: 31340143).<br>The anti-Tubulin Alpha antibody has been validated for immunoblotting in C. elegans (PMID: 30450365, 31340143, 23171715).<br>The anti-GFP antibody has been validated for immunoblotting in C. elegans (PMID: 34172445).

Animals and other research organisms

Policy information about studies involving animals; ARRIVE guidelines recommended for reporting animal research, and Sex and Gender in Research

Laboratory animals	Organism: Caenorhabditis elegans.<br>Strains: wild-type Bristol N2, AM140 rmIs132[unc-54p::Q35::YFP] I, CB1370 daf-2(e1370) III, DA465 eat-2(ad465) II, DCL4 rsks-1 (ok1255) III, DCL29 acs-20(tm3232) IV, DCL33 eat-2(ad465) II; acs-20(tm3232) IV, DCL58 eat-2(ad465) II; rde-1(ne219) V; kzIs9 [lin-26p::rde-1 + pRF4], DCL59 eat-2(ad465) II; rde-1(ne300) V; neIs9[myo-3p::HA::RDE-1 + pRF4] X, DCL90 rsks-1(ok1255) III; acs-20 (tm3232) IV, DCL201 glp-1(e2144) III, DCL285 ptr-8(mkc18) II, DCL288 ptr-8(mkc18) eat-2(ad465)II, DCL289 ptr-8(mkc18) eat-2 (ad465) II; acs-20(tm3232) IV, DCL290 ptr-8(mkc18) II; acs-20(tm3232) IV, DCL545 acs-20[mkc31(acs-20::Degron::GFP::3 x Flag)] IV, DCL648 acs-20(tm3232) IV; mkcSi36[dpy-7p::acs-20::SL2::GFP], DCL663 mkcIs20[ptr-8p::GFP + pCFJ90] IV, DCL679 eat-2(ad465) II; acs-20(tm3232) IV; mkcSi36[dpy-7p::acs-20::SL2::GFP], DCL722 eat-2(ad465) II; mkcIs20[ptr-8p::GFP + pCFJ90] IV, DCL800 rmIs132 [unc-54p::Q35::YFP] I; eat-2(ad465) II, DCL801 rmIs132[unc-54p::Q35::YFP] I; acs-20(tm3232) IV, DCL802 rmIs132[unc-54p::Q35::YFP] I; eat-2(ad465) II; acs-20(tm3232) IV, DCL897 nhr-23[mkc80(nhr-23::Degron::GFP::3 x Flag)] I, DCL926 mkcEx221[ptr-8 (Δ1.5-3k) p::gfp + pCFJ90], DCL927 mkcEx222[ptr-8 (Δ1.5-3k) p::gfp + pCFJ90], DCL928 mkcEx223[ptr-8 (Δ1.5-3k) p::gfp + pCFJ90], DCL929 mkcEx224[ptr-8 (Δ1.5-3k) p::gfp + pCFJ90], DCL939 mkcSi80[col-12p::GFP::DGAT-2::let-858 3'UTR + unc-119(+)] II, DCL941 isp-1 (qm150) acs-20(tm3232) IV, DCL946 daf-2(e1370) III; acs-20(tm3232) IV, DCL991 mkcSi80[col-12p::GFP::DGAT-2::let-858 3'UTR + unc-119(+)] eat-2(ad465) II, DCL992 mkcSi80[col-12p::GFP::DGAT-2::let-858 3'UTR + unc-119(+)] II; acs-20(tm3232) IV, DCL993 mkcSi80[col-12p::GFP::DGAT-2::let-858 3'UTR + unc-119(+)] eat-2(ad465) II; acs-20(tm3232) IV, DCL1023 mkcSi98 [col-12p::rde-1::col-12 3'UTR + unc-119(+)] II; rde-1(mkc36) V, DCL1024 eat-2(ad465) mkcSi98 [col-12p::rde-1::col-12 3'UTR + unc-119(+)] II; rde-1(mkc36) V, DCL1027 mkcEx239[ptr-8 (Δ1.5-1.8k)p::gfp + pCFJ90], DCL1028 mkcEx240[ptr-8 (Δ1.5-1.8k)p::gfp + pCFJ90], DCL1029 mkcEx241[ptr-8 (Δ1.9-2.7k)p::gfp + pCFJ90], DCL1030 mkcEx242[ptr-8 (Δ1.9-2.7k)p::gfp + pCFJ90], DCL1031 mkcEx243[ptr-8 (Δ2.7-3k)p::gfp + pCFJ90], DCL1032 mkcEx244[ptr-8 (Δ2.7-3k)p::gfp + pCFJ90], DCL1059 ptr-8[mkc109(Δ1.9-2.7 k)] II, DCL1060 ptr-8[mkc109(Δ1.9-2.7 k)] eat-2(ad465) II, DCL1228 eat-2(ad465) II; hjsi56[vha-6p::3×FLAG-TEV-GFP::dgat-2::let-858 3'UTR] IV, DCL1229 hjsi56[vha-6p::3×FLAG-TEV-GFP::dgat-2::let-858 3'UTR] acs-20(tm3232) IV, DCL1230 eat-2(ad465) II; hjsi56 [vha-6p::3×FLAG-TEV-GFP::dgat-2::let-858 3'UTR] acs-20(tm3232) IV, MQ887 isp-1(qm150) IV, NR222 rde-1(ne219) V; kzIs9 [lin-26p::rde-1 + pRF4], VS29 hjsi56[vha-6p::3×FLAG-TEV-GFP::dgat-2::let-858 3'UTR] IV, WM118 rde-1(ne300) V; neIs9 [myo-3::HA::RDE-1 + pRF4] X.<br>Stage:L2, L3, L4, day 1, day 3, day 4 adults, whole life (lifespan experiments)
Wild animals	No wild animals were used in this study.
Reporting on sex	All experiments were carried out using hermaphrodites.
Field-collected samples	No field-collected samples were used in this study.
Ethics oversight	No ethical approval or guidance is required for research using C. elegans.

Note that full information on the approval of the study protocol must also be provided in the manuscript.
